# Coordination of photosynthetic performance and leaf N:P stoichiometry under fluctuating coastal environmental conditions in *Bolboschoenoplectus mariqueter*

**DOI:** 10.3389/fpls.2026.1911028

**Published:** 2026-10-12

**Authors:** Jiayuan Wang, Lianjiang Lyu, Xuexin Shao, Tonggui Wu, Fang Zhou, Long Zhang, Wenwen Yuan

**Affiliations:** 1 Shaoxing Municipal Natural Resources Protection and Management Center, Shaoxing, Zhejiang, China; 2 Shaoxing Municipal Urban and Rural Planning Management Center, Shaoxing, Zhejiang, China; 3 State Key Laboratory of Wetland Conservation and Restoration, Wetland Ecosystem Research Station of Hangzhou Bay, Research Institute of Subtropical Forestry, Chinese Academy of Forestry, Hangzhou, China

**Keywords:** × Bolboschoenoplectus mariqueter, factors, leaf stoichiometry, photosynthesis, transpiration

## Abstract

**Introduction:**

The population of *Bolboschoenoplectus mariqueter* has substantially declined because of habitat modification and competition. Therefore, this study aimed to characterize the photosynthetic and stoichiometric traits of *B. mariqueter* and their environmental regulation in a natural tidal-flat population to facilitate successful conservation.

**Methods:**

Light- and CO_2_-response characteristics, diurnal and seasonal dynamics of leaf gas exchange, daily carbon assimilation and transpiration, chlorophyll characteristics, and leaf nitrogen (N) and phosphorus (P) stoichiometry in a natural were investigated in a × *B. mariqueter* population in Hangzhou Bay, China. Random forest, variation partitioning, and redundancy analyses were used to evaluate the relationships among photosynthetic performance, leaf nutrient stoichiometry, and microenvironmental factors.

**Results:**

The net photosynthetic rate (Pn), transpiration rate (Tr), and water-use efficiency (WUE) varied substantially among months. Pn and Tr showed generally coordinated diurnal dynamics, whereas daily carbon assimilation and transpiration followed partially divergent seasonal trajectories after July. Leaf N and P concentrations and N:P ratios exhibited pronounced and asynchronous seasonal variations. The random forest model explained 65%, 69%, and 42% of the variations in Pn, Tr, and WUE, respectively. Leaf P and N:P were the main predictors of Pn; N, P, and N:P, of Tr; and N and P, of WUE. Variation partitioning showed substantial shared explanatory variation among photosynthetic-pigment traits, leaf nutrient stoichiometry, and microenvironmental factors, whereas redundancy analysis (RDA) identified leaf N, P, N:P, leaf temperature (LT), vapor pressure deficit (VPD), and chlorophyll a/b (Chl a/b) as key explanatory variables.

**Conclusion:**

These results indicate that photosynthetic performance and leaf nutrient stoichiometry in *B. mariqueter* are linked within an integrated environmental–physiological framework rather than through a simple direct relationship between leaf nutrient concentrations and carbon assimilation. Thus, P availability may play an important role in seasonal nutrient regulation. This multidimensional coordination of carbon acquisition, water use, and nutrient status may contribute to the adaptation of *B. mariqueter* to the dynamic coastal tidal-flat environment and provide a physiological basis for its conservation and restoration.

## 1 Introduction

*× Bolboschoenoplectus mariqueter* is a perennial pioneer species widely distributed in the intertidal wetlands of the Yangtze River Estuary and Hangzhou Bay of China, where it contributes to sediment stabilization, carbon sequestration, and habitat provision for wetland fauna (Wu et al., 2008a; Zhong et al., 2020; Gong et al., 2023). However, its population has substantially declined because of habitat modification and competition with the invasive *Spartina alterniflora* (Chen et al., 2004). Therefore, successful conservation and restoration of *B. mariqueter* require a mechanistic understanding of the physiological processes that regulate its growth and adaptation to a highly dynamic tidal-flat environment. Photosynthetic carbon assimilation directly determines plant carbon gain and biomass production, whereas leaf nitrogen (N) and phosphorus (P) availability regulates photosynthetic machinery, metabolic activity, and plant growth. Leaf N:P stoichiometry can reflect the relative balance of N and P supply and has been widely used as an indicator of potential nutrient limitation (Koerselman and Meuleman, 1996). Examining photosynthetic traits together with N:P stoichiometry may, therefore, provide an integrated perspective on the carbon–nutrient relationships underlying the growth and environmental adaptation of *B. mariqueter*.

The coupling between photosynthetic performance and nutrient status may be particularly important for *B. mariqueter* because tidal-flat plants experience simultaneous fluctuations in light, temperature, atmospheric evaporative demand, salinity, inundation, and nutrient availability. Excessive salinity, prolonged flooding, and extremely high temperatures suppress the growth and photosynthetic performance of *B. mariqueter* (Li et al., 2018, 2019; Hu et al., 2020; Liu et al., 2020), whereas nutrient availability can substantially modify its growth response (Zhang et al., 2017; Wei, 2019). Photosynthetic carbon gain depends not only on irradiance but also on stomatal regulation and plant water balance. In particular, increasing vapor pressure deficit (VPD) can enhance transpirational water loss and induce stomatal limitation, potentially creating a tradeoff between carbon assimilation and water conservation (Hsu et al., 2021; Diao et al., 2024a). N is closely associated with photosynthetic proteins and enzymes, whereas P supports energy metabolism and growth, and imbalances in N and P availability may therefore constrain photosynthetic capacity and biomass accumulation. Consequently, integrating seasonal and diurnal photosynthetic dynamics with leaf N:P stoichiometry can help to clarify how carbon assimilation, water regulation, and nutrient limitation jointly influence the growth and stress resistance of *B. mariqueter*. Nevertheless, these processes have rarely been evaluated together in natural tidal flats.

To address this knowledge gap, this study investigated a natural *B. mariqueter* population in the Hangzhou Bay coastal wetland and characterized its light- and CO_2_-response traits, diurnal and seasonal dynamics of photosynthesis and transpiration, water use efficiency, chlorophyll content, leaf N:P stoichiometry, and their relationships with environmental factors. By integrating photosynthetic physiology with nutrient stoichiometry, this study aimed to identify the environmental and nutritional conditions associated with favorable physiological performance, thereby providing a physiological basis for seedling cultivation and community restoration. Specifically, our objectives were to (1) elucidate the regulatory patterns of growth and stress resistance in *B. mariqueter* in relation to photosynthetic traits, environmental conditions, and N:P stoichiometry, and to identify favorable ranges supported by field observations; and (2) propose practical recommendations for promoting the restoration of *B. mariqueter* communities.

## 2 Materials and methods

### 2.1 Study site

The study was conducted at the National Forestry and Grassland Administration Hangzhou Bay Wetland Ecosystem Research Station, Zhejiang Province, China (120°08′43″E, 30°18′40″N). The site had a mean annual temperature of 16 °C, mean annual sunshine duration of 2038.4 h, and a frost-free period of 244 d. Mean annual precipitation was 1344.7 mm. The tidal flat was strongly influenced by tides, with a semidiurnal, reversing current regime; the flood tide lasted 6 h, the ebb tide 6.4 h, and the current direction was nearly parallel to the coastline. The soil was a coastal soil formed by sediment deposition from the Yangtze River and local rivers. Dominant vegetation included *×B. mariqueter* and *Phragmites australis* (Wu et al., 2008b).

### 2.2 Sampling plot

Three 30 m × 30 m permanent plots with relatively uniform *×B. mariqueter* stands were established along a north–south transect perpendicular to the coastline, with 5 m spacing between plots. Each plot was divided into nine 1 m × 1 m quadrats. Three 0.5 m × 0.5 m subplots were randomly selected within each quadrat. After photosynthetic measurements, intact mature leaves were collected using the quartering method, oven-dried at 80 °C to constant weight, ground, sieved, and stored for subsequent determination of chlorophyll content and leaf N and P contents.

### 2.3 Physiological trait measurements

#### 2.3.1 Photosynthetic measurements

From May to November, on two to three clear days in the latter part of each month, measurements were taken between 8:00 and 18:00 using a LI-6400 portable photosynthesis system (LICORbio, Lincoln, NE, United States USA). Three to five plants were selected, and five to seven mature, intact functional leaves per plant were measured, with three replicates per measurement. The net photosynthetic rate (Pn), stomatal conductance (Gs), ambient CO_2_ concentration (Ca), VPD, leaf temperature (Ti), relative humidity (RH), and photosynthetically active radiation (PAR) were recorded.

#### 2.3.2 Intrinsic water-use efficiency

At the leaf level, instantaneous water-use efficiency (WUE) was calculated as the ratio of net photosynthetic rate to transpiration rate (Pn/Tr). This metric represents the instantaneous balance between carbon assimilation and transpirational water loss and differs from intrinsic water-use efficiency (iWUE), which is commonly expressed as Pn/Gs.

#### 2.3.3 Light and CO_2_ response curves

During the peak growth period (July–August) on clear days between 8:00 and 10:30 (when leaves were fully activated and physiologically active), five plants with similar growth statuses were selected. Light response curves were measured using the LI-6400 (LICORbio) red–blue light source with an air flow rate of 0.5 L·min^-1^, temperature controlled at 37 ± 1 °C, relative humidity at approximately 60%, and CO_2_ concentration at 365 μmol·mol^-1^. Pn was measured sequentially at photosynthetic photon flux density (PPFD) levels of 2500, 2200, 2000, 1800, 1500, 1200, 1000, 800, 600, 400, 200, 100, 50, 20, and 0 μmol·m^-2^·s^-1^. For the CO_2_ response curves, the plants were induced under saturated light for 30 min. After photosynthesis reached a steady state, the CO_2_ concentration in the reference chamber was controlled using a LI-6400-01 (LICORbio) liquefied CO_2_ cylinder. Pn was measured sequentially at CO_2_ concentrations (Ci) of 300, 250, 200, 150, 100, and 50 μmol·mol^-1^; the was then returned to 300 μmol·mol^-1^, and after stabilization, measured at 400, 600, 800, 1000, 1200, 1500, and 2000 μmol·mol^-1^.

#### 2.3.4 Chlorophyll content

Chlorophyll was extracted using the acetone–ethanol method (Li et al., 2006). Absorbance was measured at 645 and 663 nm using a 723N visible spectrophotometer. Chlorophyll a (Chl a) and chlorophyll b (Chl b) were calculated using the following Equations 1, 2:

(1) $$Chl\;a=\frac{12.7\times A663-2.69\times A645}{FW}\times V$$

(2) $$Chl\;b=\frac{22.9\times A645-4.68\times A633}{FW}\times V$$

where *V* is the extraction volume (mL), and *FW* is the fresh weight (g). Total chlorophyll was obtained as their sum.

#### 2.3.5 Leaf stoichiometric traits

Leaf samples were oven-dried to a constant mass, ground to a fine powder, and passed through a sieve prior to chemical analysis. To determine the total N content, an appropriate amount of dried leaf powder was digested with concentrated sulfuric acid (H_2_SO_4_) in a temperature-controlled digestion system until the digest became clear. After cooling, the digest was diluted to a known volume with deionized water and the total N concentration was determined using the Kjeldahl method. For total P determination, dried leaf samples were digested at 375 °C, and the resulting digest was quantitatively transferred to a volumetric flask and diluted to a known volume. Phosphorus concentration was then determined using the molybdenum–antimony–ascorbic acid colorimetric method. Briefly, the orthophosphate in the digest was reacted with ammonium molybdate and antimony reagent under acidic conditions and reduced with ascorbic acid to form a blue phosphomolybdate complex, which was quantified spectrophotometrically against a phosphate standard curve. Leaf N and P concentrations were expressed on a dry-mass basis (mg·g^-1^), and the leaf N:P ratio was calculated on a mass basis.

### 2.4 Data processing and analysis

Light response curves were plotted with PPFD as the abscissa and Pn as the ordinate and fitted using the non-rectangular hyperbola model (Equation 3; Herrick and Thomas, 1999):

(3) $$P_{n}=\frac{AQY\cdot PPFD+P_{max}-\sqrt{{\left(AQY\cdot PPFD+P_{max}\right)}^{2}-4\theta \cdot AQY\cdot P_{max}\cdot PPFD}}{2\theta }-R_{d}$$

where *Pn* is the net photosynthetic rate (μmol·m^-2^·s^-1^), *AQY* is the apparent quantum yield (mol·mol^-1^), *Pmax* is the maximum net photosynthetic rate (μmol·m^-2^·s^-1^), *PPFD* is the photosynthetic photon flux density (μmol·m^-2^·s^-1^), *θ* is the convexity of the light response curve, and *Rd* is the dark respiration rate (μmol·m^-2^·s^-1^). Photosynthetic parameters, including AQY, light saturation point (LSP), and light compensation point (LCP) were derived using SPSS fitting.

For the CO_2_ response curve, Pn and Ci values with Ci < 200 μmol·mol^-1^ were used to construct a linear regression, the slope of which represented the CO_2_ carboxylation efficiency (CCE). The CO_2_ compensation point (CCP) was also calculated (Meng et al., 2007). Daily assimilation and daily transpiration were calculated following Luo et al. (2007).

Random forest analysis was used to evaluate the relative importance of physiological, leaf stoichiometric, and microenvironmental variables in explaining variation in Pn, Tr, and WUE. Variation partitioning analysis was performed to partition the independent and shared explanatory fractions attributable to photosynthetic pigment traits, leaf nutrient stoichiometry, and microenvironmental factors. Redundancy analysis (RDA) was conducted to characterize the multivariate relationships between photosynthetic performance and the explanatory variables. The significance of explanatory variables in the RDA was evaluated using permutation tests.

## 3 Result

### 3.1 Photosynthetic characteristics of *B. mariqueter*

#### 3.1.1 Light and CO_2_ response characteristics

The Pn exhibited a typical saturation response with increasing PPFD (**Figure 1A**). Pn increased rapidly under low-to-moderate irradiance and gradually approached saturation at higher PPFD. The fitted light-response curve yielded an apparent quantum efficiency (AQE) of 0.051 ± 0.003 mol·mol^-1^, a Pmax of 28.54 ± 2.87 μmol·m^-2^·s^-1^, and an Rd of 2.22 ± 0.18 μmol·m^-2^·s^-1^. The fitted LCP and LSP were 36.76 ± 2.28 μmol·m^-2^·s^-1^ and 571.58 ± 25.53 μmol·m^-2^·s^-1^, respectively.

**Figure 1:**
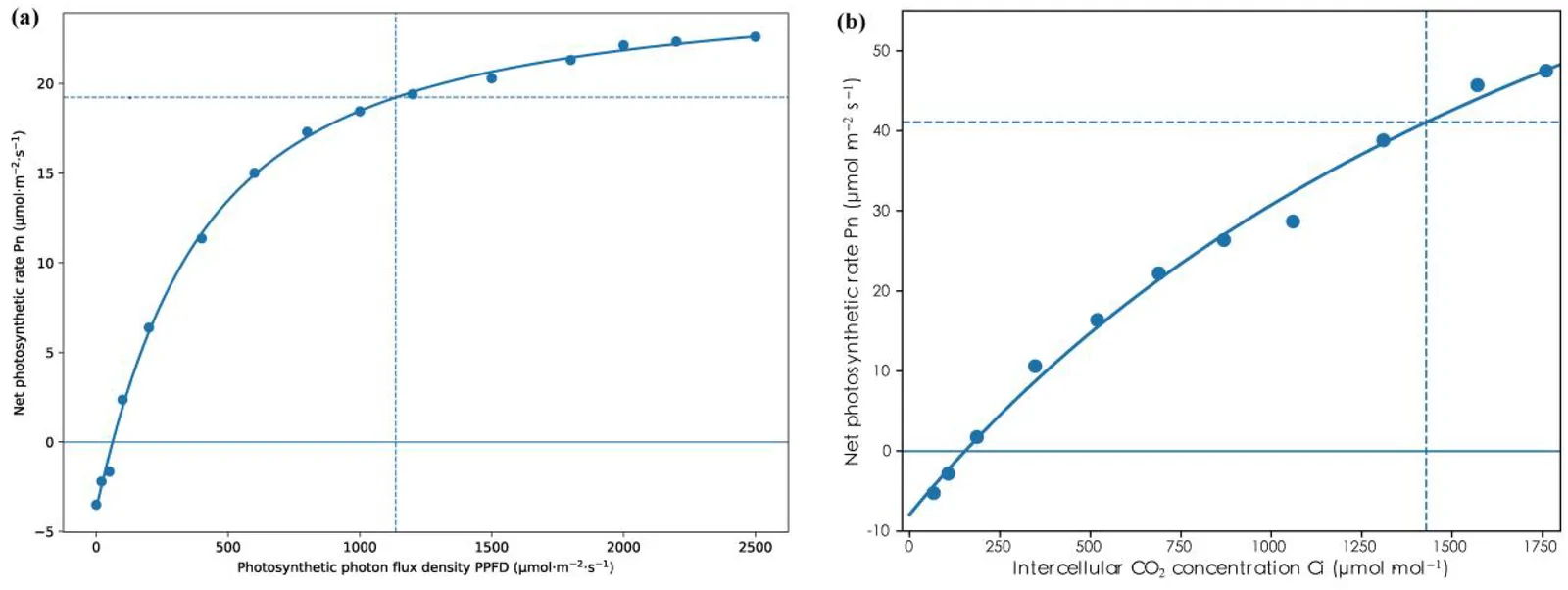
Photosynthetic light- and CO_2_ -response curves of *B. mariqueter.* **(a)** Response of net photosynthetic rate (Pn) to photosynthetic photon flux density (PPFD); **(b)** response of Pn to intercellular CO_2_ concentration (Ci). Points represent observed values and solid curves represent fitted response functions. Dashed lines indicate the estimated saturation values and corresponding PPFD or Ci derived from the fitted curves.

Pn also increased with Ci, though the rate of increase gradually declined at higher Ci (**Figure 1B**). The fitted CO_2_-response curve yielded a CCE of 0.057 ± 0.002, and the CCP was 153.43 ± 11.6 μmol·mol^-1^.

#### 3.1.2 Seasonal and diurnal dynamics of photosynthetic traits

The photosynthetic traits of *B. mariqueter* varied substantially during the growing season (**Table 1**). The Pn showed significant monthly variation (F = 8.76, p < 0.001), with values in July being significantly higher than those in May, September, and October (p < 0.05). The Tr also differed significantly among months (F = 5.34, p < 0.05), with values in July being significantly higher than those in May and October (p < 0.05). WUE varied significantly among months (F = 3.21, p < 0.05), with values in July being significantly higher than those in May and October (p < 0.05).

**Table 1:** Significance analysis of photosynthetic parameters among different months.

Parameter	F value	*p* value	Multiple comparison (LSD)
Net photosynthetic(Pn)	8.76	< 0.001	Jul. > May.*, Jul. > Sep.*, Jul. > Oct.*
Transpiration(Tr)	5.34	< 0.05	Jul. > May.*, Jul. > Oct.*
Water use efficiency(WUE)	3.21	< 0.05	Jul. > May.*, Jul. > Oct.*

*indicates significant differences (p < 0.05); data are based on repeated measurements from three plots (n = 9).

The diurnal dynamics of Pn, Tr, and WUE differed markedly among months (**Figure 2**). Pn generally increased or remained relatively high from late morning to early afternoon and subsequently declined toward late afternoon, although the timing and magnitude of the daily maxima differed among the months (**Figure 2A**). The Tr exhibited broadly similar diurnal dynamics, with relatively high values in the morning and midday, followed by an overall decline in the afternoon (**Figure 2B**). In contrast, WUE showed greater temporal variability and was not consistently synchronized with either Pn or Tr (**Figure 2C**).

**Figure 2:**
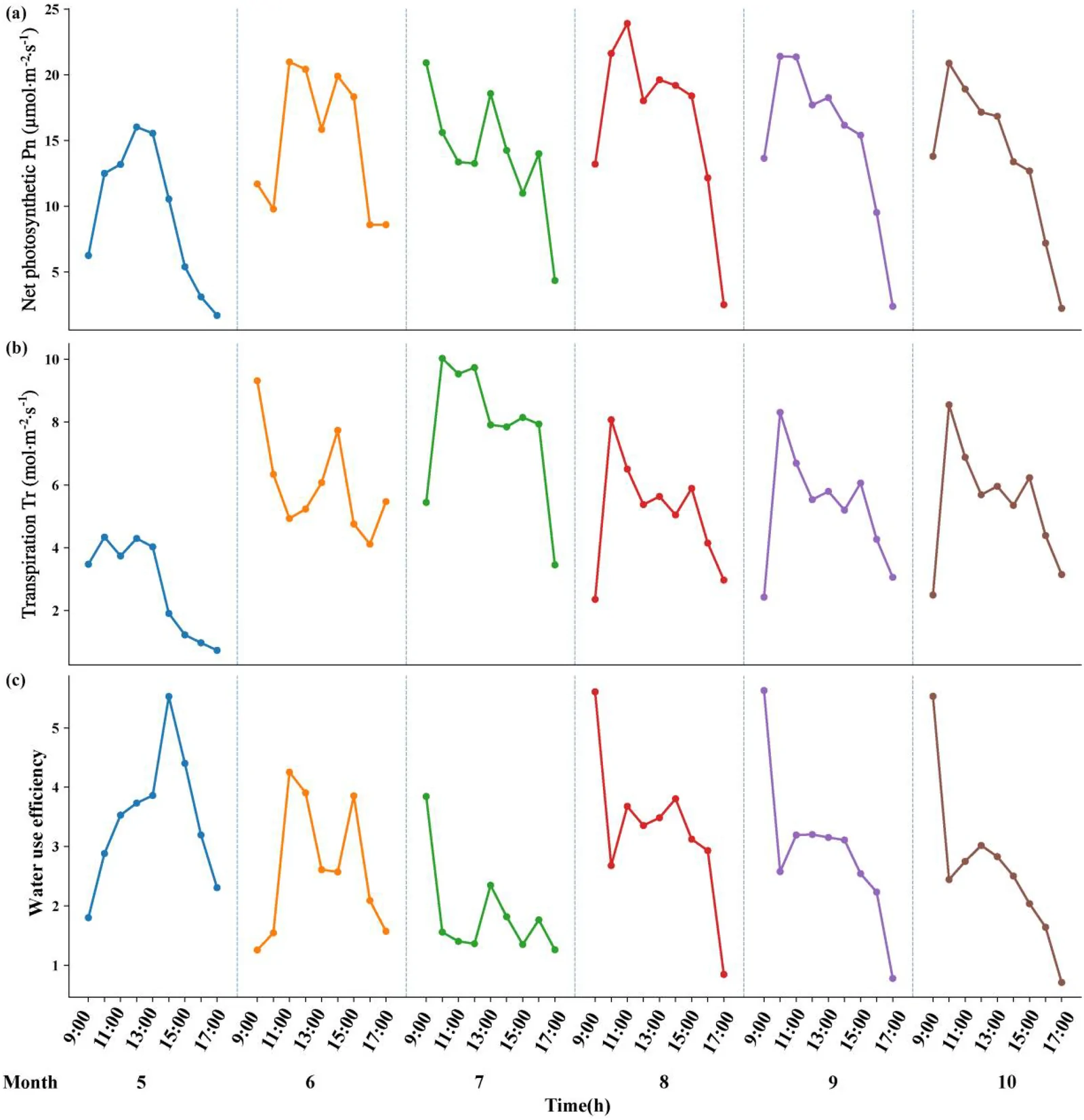
Diurnal dynamics of net photosynthetic rate, transpiration rate, and water-use efficiency of B. mariqueter during the growing season (May–October). **(a)** Net photosynthetic rate (Pn), **(b)** transpiration rate (Tr), and **(c)** water-use efficiency (WUE). Values are presented as mean SE based on repeated measurements from three plots (n = 9).

Daily integrated carbon assimilation and transpiration exhibited distinct seasonal trajectories (**Figure 3**). Both parameters increased from May to the mid-growing season. Daily carbon assimilation increased markedly from May to June and remained relatively high during July–August, before declining during September–October. In contrast, daily transpiration reached its highest level in July and subsequently decreased markedly from August onward. Thus, daily carbon assimilation and transpiration showed broadly coordinated changes during the early growing season but partially divergent seasonal trajectories after July.

**Figure 3:**
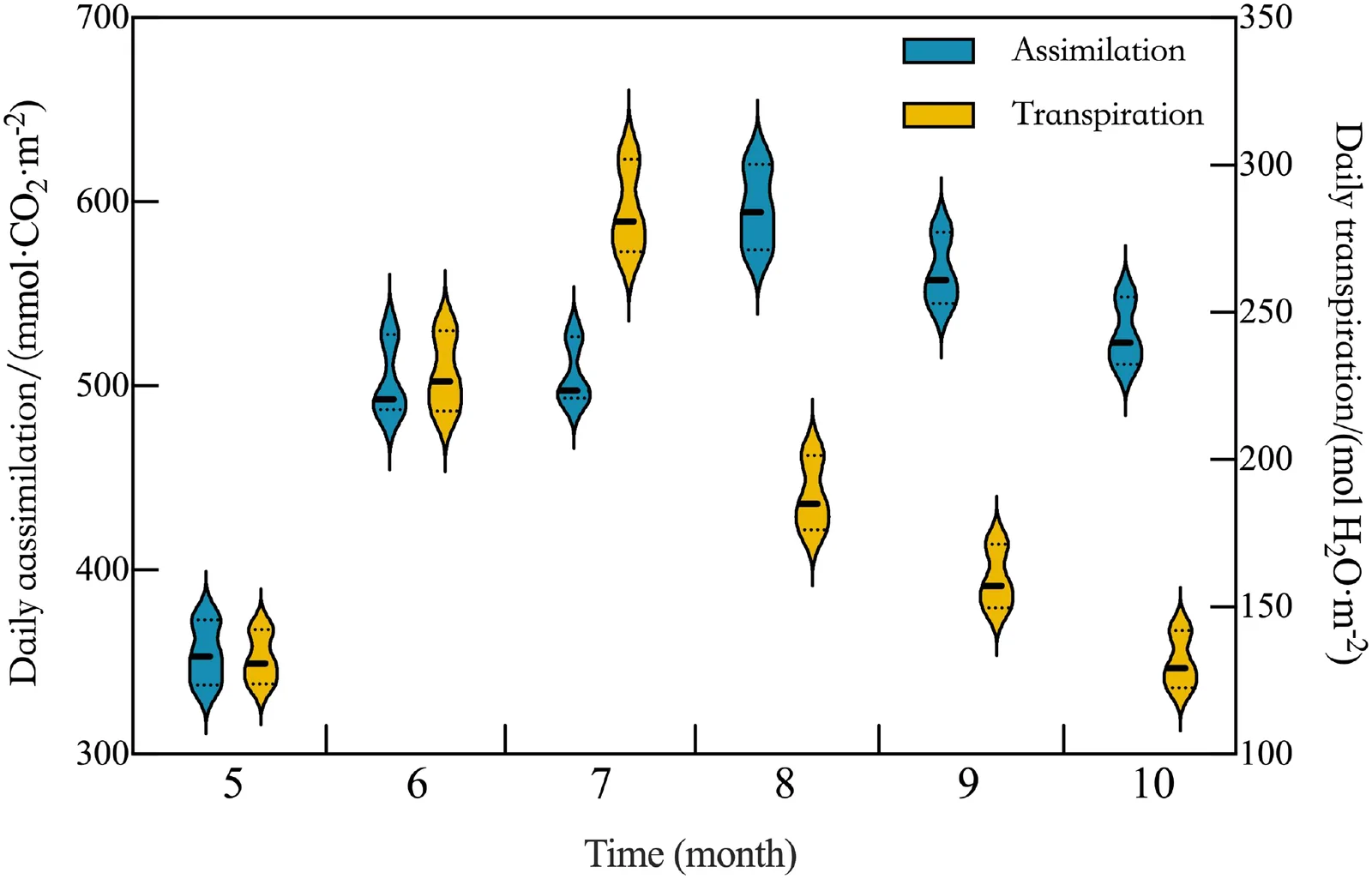
Seasonal variation in daily carbon assimilation and transpiration of B. mariqueter during the growing season (May–October). Blue violins represent daily carbon assimilation and correspond to the left y-axis, whereas yellow violins represent daily transpiration and correspond to the right y-axis. The thick solid line within each violin indicates the median, and the lower and upper dotted lines indicate the 25th (Q1) and 75th (Q3) percentiles, respectively. Data were obtained from repeated measurements across three plots (n = 9).

#### 3.1.3 Seasonal dynamics of chlorophyll and leaf nutrient stoichiometry

Leaf Chl a and Chl b showed distinct dynamics during the growing season (**Figure 4**). Chl a was relatively low in May and June, increased markedly in July, and remained high from August to October. In contrast, the Chl b levels decreased from May to June, increased markedly in July, and subsequently declined to relatively low levels from August to October. Correspondingly, the relative contribution of Chl a to the total chlorophyll increased during the middle and later stages of the growing season, whereas that of Chl b decreased.

**Figure 4:**
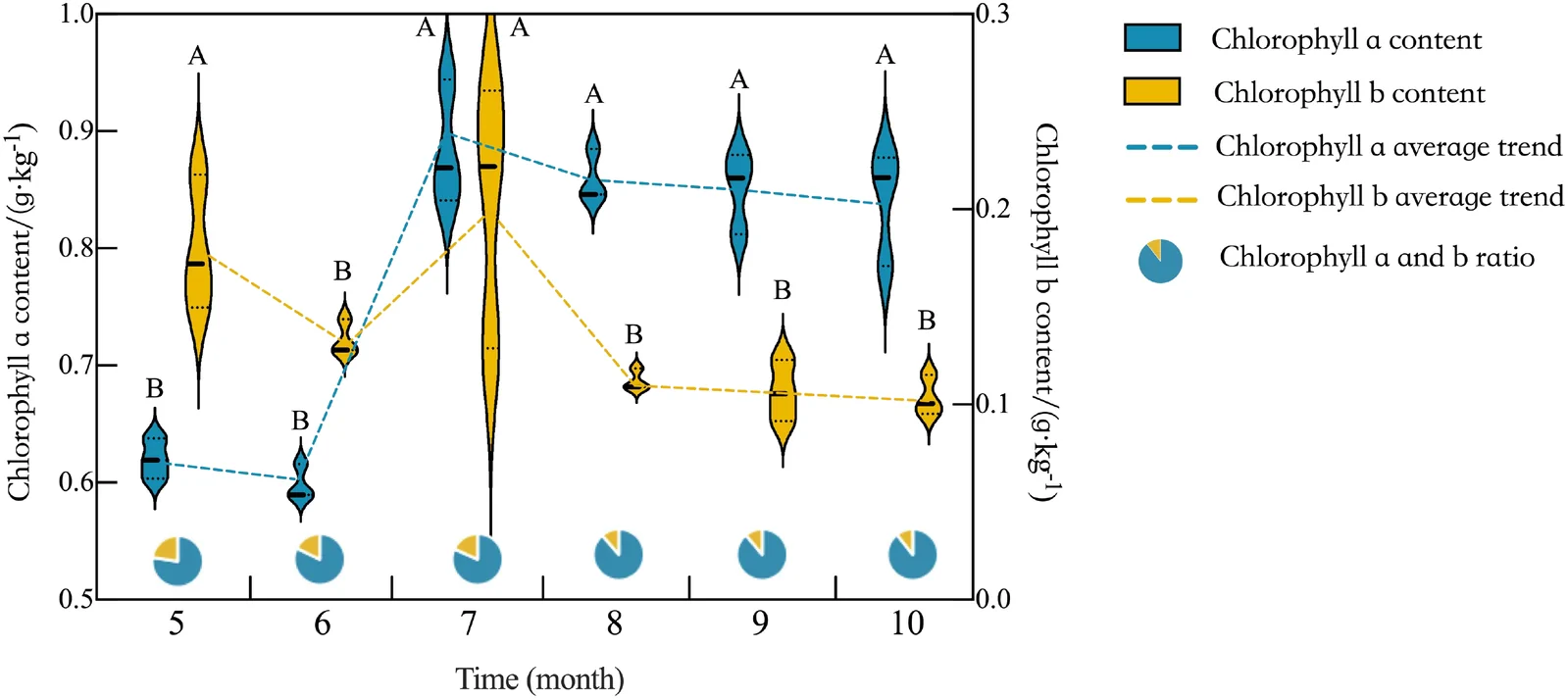
Seasonal dynamics of leaf chlorophyll content and composition in B. mariqueter during the growing season (May–October). Blue and yellow violins represent Chl a and Chl b contents, respectively. Dashed lines connect the monthly median values, and pie charts show the relative proportions of Chl a and Chl b in total chlorophyll. The thick solid line within each violin indicates the median, whereas the lower and upper dotted lines indicate the 25th (Q1) and 75th (Q3) percentiles, respectively. Data were obtained from repeated measurements across three plots (n = 9).

Leaf N and P concentrations and the N:P ratio differed significantly among months (p < 0.05; **Figure 5**). Leaf N concentration was the highest in May and was significantly higher than that in all other months (p < 0.05). It declined to its lowest level in July and subsequently increased, with September reaching the second highest level (**Figure 5A**). The leaf P concentration was also significantly higher in May than in the other months (p < 0.05). Following a sharp decline to its lowest level in June, the P concentration increased from July to September, with significantly higher values in August and September than in June, before declining again in October (p < 0.05; **Figure 5B**). The N:P ratio exhibited a contrasting seasonal pattern, increasing from its lowest level in May to its highest level in June. Thereafter, the N:P ratio decreased and remained at intermediate levels from July to October (**Figure 5C**).

**Figure 5:**
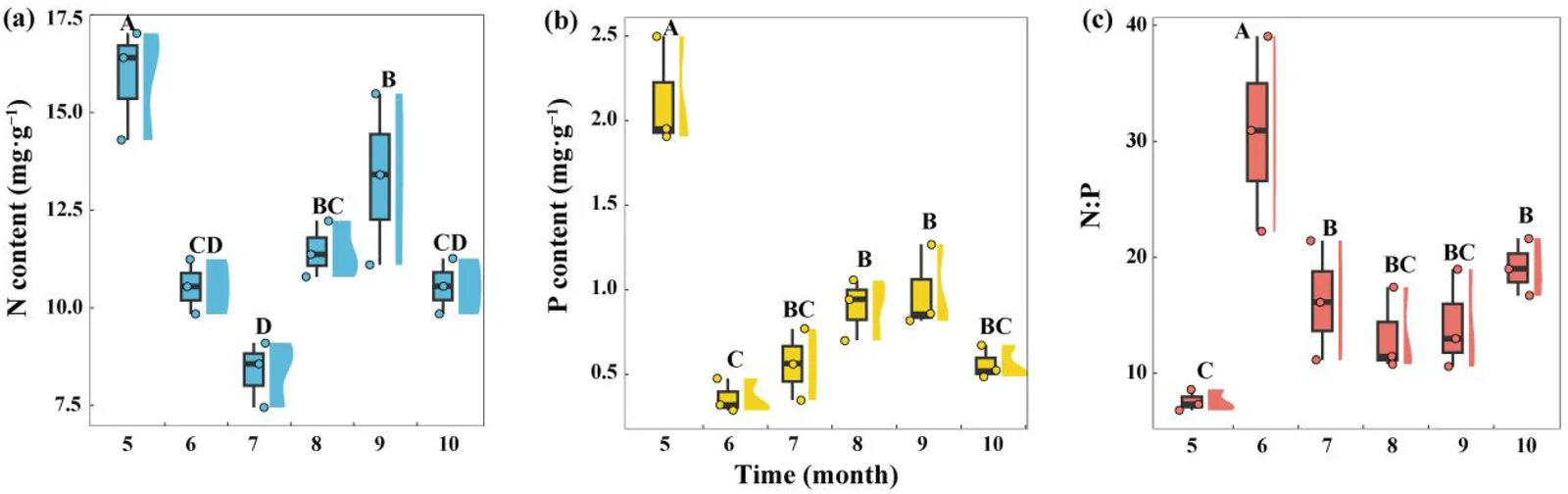
Seasonal variation in leaf nitrogen (N) and phosphorus (P) concentrations and N:P ratio of *B. mariqueter* during the **(a)** Leaf N concentration; **(b)** leaf P concentration; and **(c)** leaf N:P ratio. Different uppercase letters indicate significant differences among months (p < 0.05).growing season (May–October).

### 3.2 Environmental regulation and coupling of photosynthetic and stoichiometric traits

#### 3.2.1 Diurnal and seasonal dynamics of microenvironmental factors

The microenvironmental factors exhibited clear diurnal and seasonal variations during the growing season (**Figure 6**). PAR generally increased in the morning, reached relatively high levels around midday, and declined in the late afternoon. Temperature showed a broadly similar pattern, although its daily maximum generally occurred later than that of PAR. RH showed an approximately opposite diurnal pattern, decreasing toward midday and recovering later in the day, whereas VPD increased toward midday or early afternoon and subsequently declined. Seasonally, PAR, temperature, and VPD were generally higher during the middle of the growing season and decreased from October to November, whereas RH showed a comparatively smaller seasonal variation.

**Figure 6:**
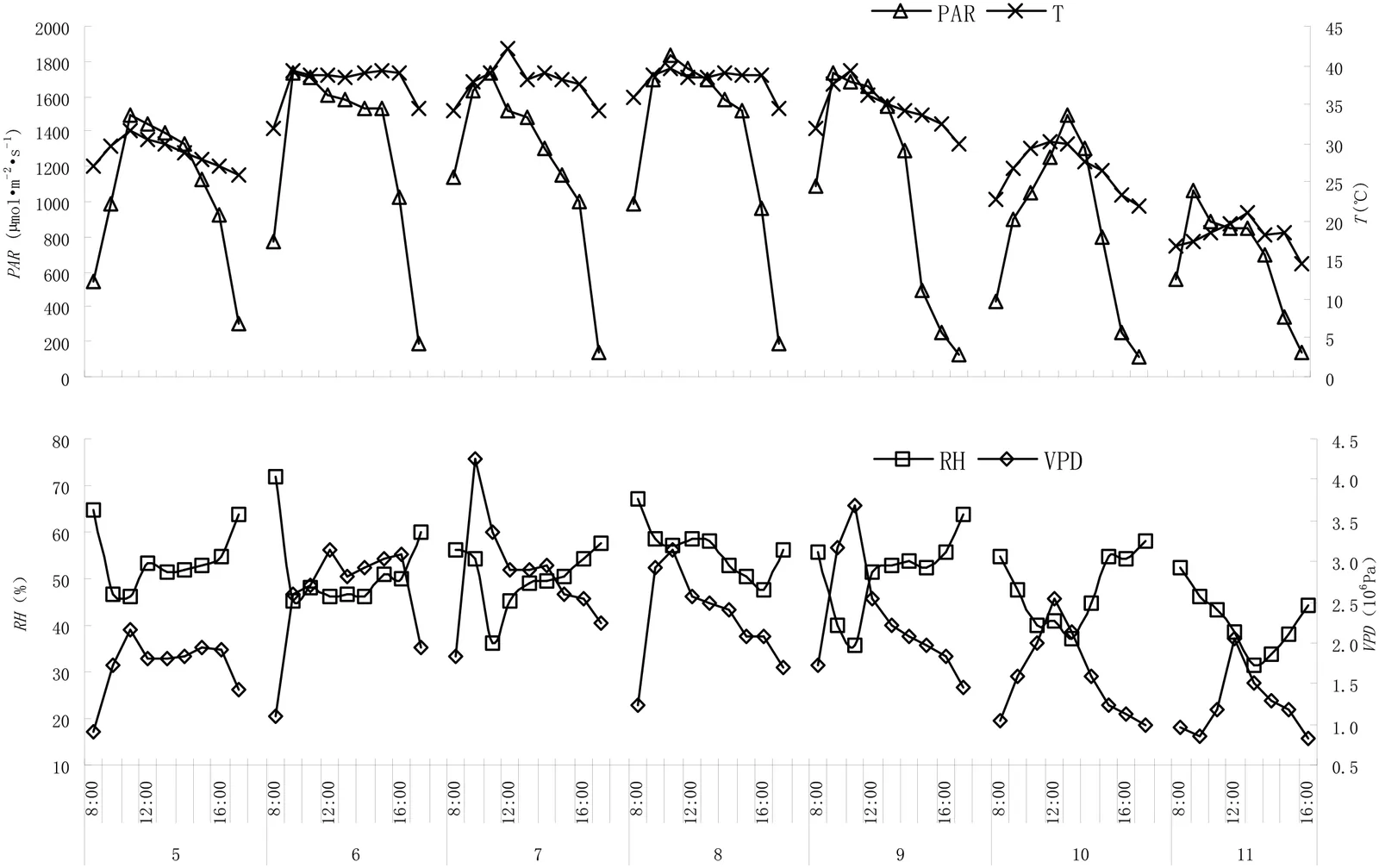
Diurnal and seasonal dynamics of microenvironmental factors during the growing season of B. mariqueter. The upper panel shows photosynthetically active radiation (PAR) and leaf temperature (Ti), and the lower panel shows relative humidity (RH) and vapor pressure deficit (VPD) from May to November. PAR, photosynthetically active radiation; RH, relative humidity; VPD, vapor pressure deficit.

#### 3.2.2 Relationships among environmental factors, photosynthetic traits, and leaf stoichiometry

Random forest analysis revealed the distinct contributions of leaf nutrient stoichiometry, microenvironmental factors, and physiological traits to photosynthetic performance (**Figure 7**). The models explained 65%, 69%, and 42% of the variation in Pn, Tr, and WUE, respectively. For Pn, leaf P concentration and N:P ratio were the two most important predictors (both p < 0.01), followed by VPD, LT, PAR, and leaf N concentration (p < 0.05; **Figure 7A**). For Tr, leaf N, P, and N:P showed the highest variable importance (p < 0.01), followed by VPD (p < 0.05; **Figure 7B**). For WUE, leaf N was the strongest predictor (p < 0.01), followed by leaf P (p < 0.05), whereas the remaining variables showed comparatively low importance (**Figure 7C**).

**Figure 7:**
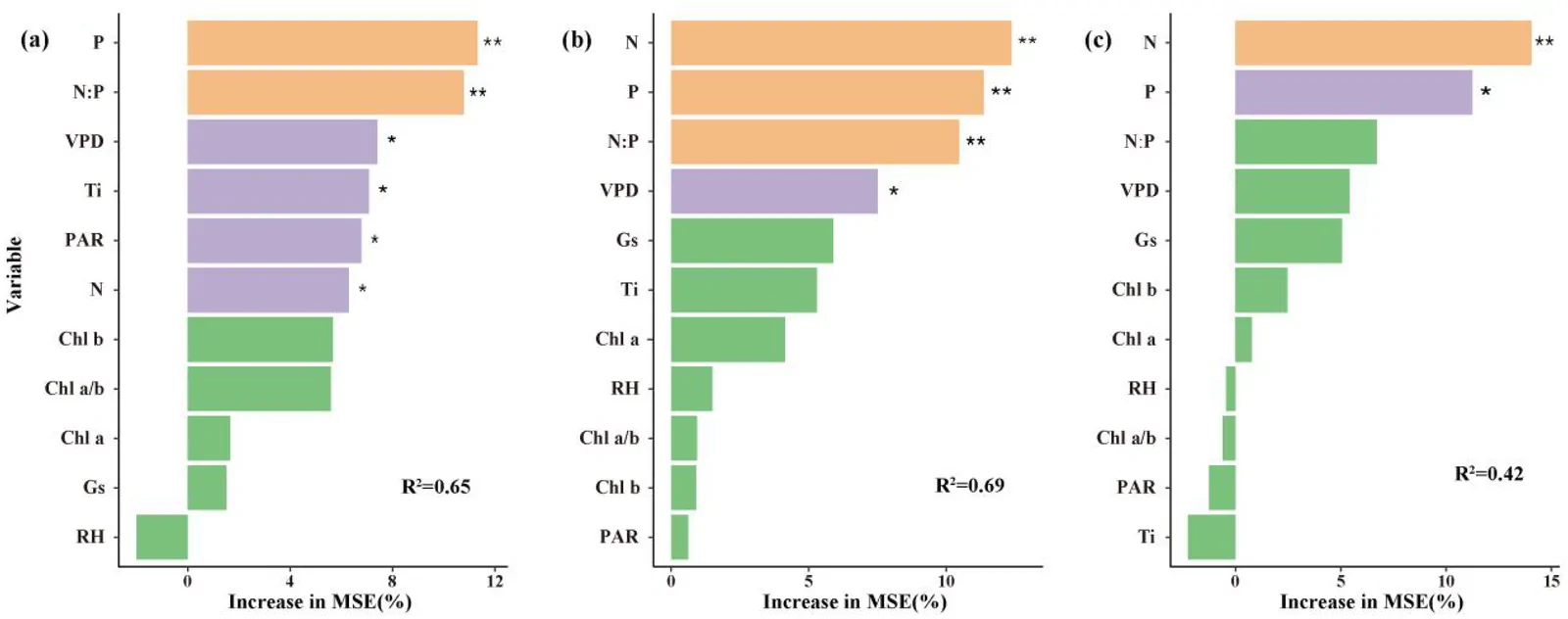
Random forest analysis of the relative importance of leaf stoichiometric, microenvironmental, and physiological variables in explaining photosynthetic performance of *B. mariqueter*. **(a)** Net photosynthetic rate (Pn), **(b)** transpiration rate (Tr), and **(c)** water-use efficiency (WUE). Variable importance is expressed as the percentage increase in mean squared error [Increase in MSE (%)]. Asterisks indicate the significance of predictor importance (p < 0.05, *p < 0.01). Model R² values are shown in each panel. N, leaf nitrogen concentration; P, leaf phosphorus concentration; N:P, leaf N:P ratio; VPD, vapor pressure deficit; PAR, photosynthetically active radiation; Ti, leaf temperature; RH, relative humidity; Gs, stomatal conductance; Chl a, chlorophyll a; Chl b, chlorophyll b; Chl a/b, chlorophyll a/b ratio.

The variation partitioning analysis further showed that photosynthetic pigment traits, leaf nutrient stoichiometry, and microenvironmental factors collectively accounted for a large proportion of the variation in photosynthetic performance (**Figure 8**). Together, these three groups explained 96.7%, 98.8%, and 91.4% of the variation in Pn, Tr, and WUE, respectively, with residual fractions of 3.3%, 1.2%, and 8.6%, respectively. Microenvironmental factors independently explained 48.9%, 13.5%, and 23.0% of the variations in Pn, Tr, and WUE, respectively, whereas photosynthetic pigment traits independently explained 7.1%, 2.8%, and 13.8%, respectively. Considerable shared explanatory fractions were observed among the three groups of predictors for all three response variables, indicating a substantial overlap in the variation associated with physiological traits, leaf nutrient stoichiometry, and microenvironmental conditions.

**Figure 8:**
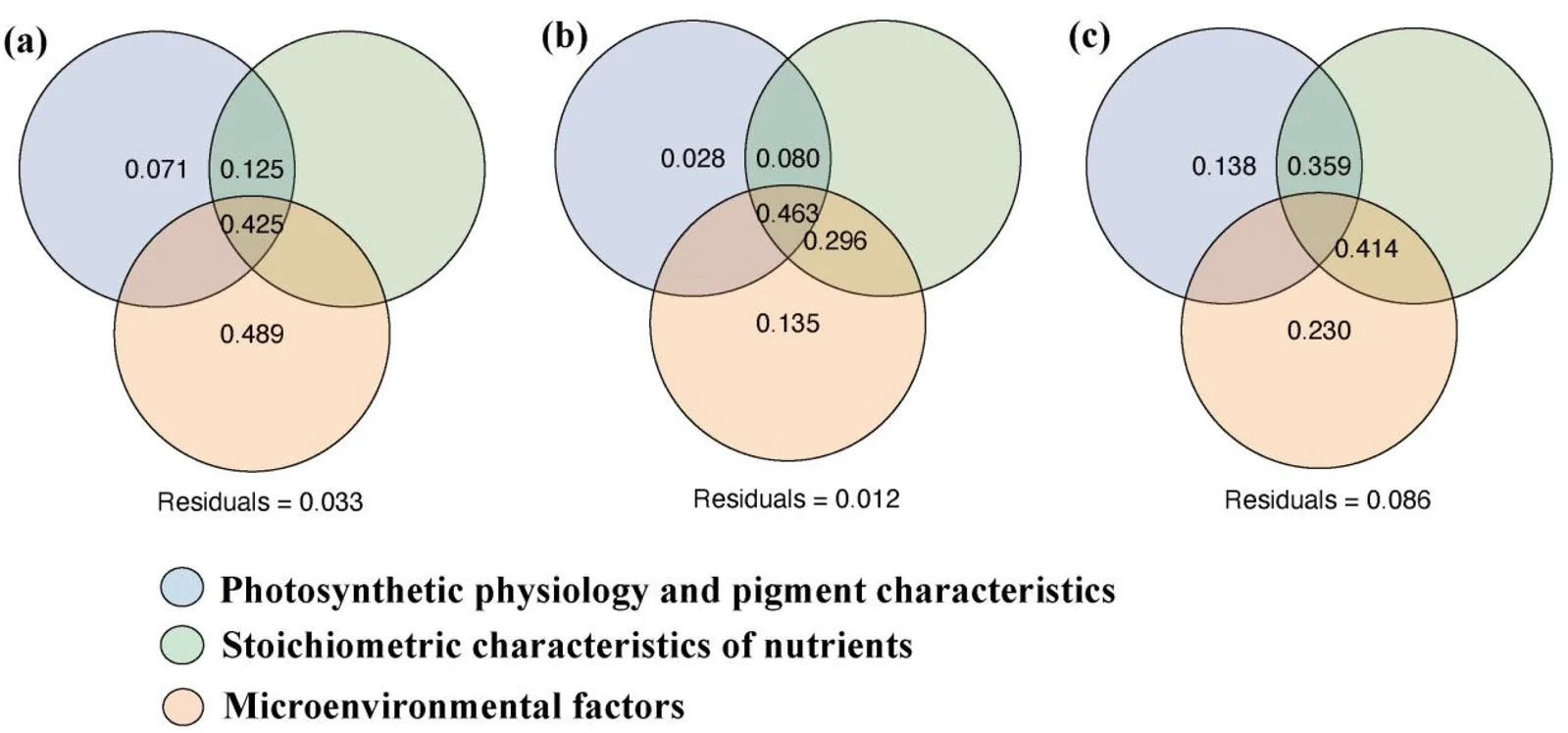
Variation partitioning analysis of photosynthetic performance explained by Photosynthetic physiology and pigment characteristics, stoichiometric characteristics of nutrients, and microenvironmental factors in *B. mariqueter.* **(a)** Net photosynthetic rate (Pn), **(b)** transpiration rate (Tr), and **(c)** water-use efficiency (WUE). Blue, green, and orange circles represent photosynthetic–pigment traits, leaf nutrient stoichiometry, and microenvironmental factors, respectively. Values within individual and overlapping fractions represent the proportions of variation explained independently or jointly by the corresponding groups of variables. Residuals represent the unexplained fractions of variation. Photosynthetic–pigment traits include Gs, Chl a, Chl b, and Chl a/b; leaf nutrient stoichiometry includes N, P, and N:P; and microenvironmental factors include PAR, Ti, RH, and VPD. Gs, stomatal conductance; Chl a, chlorophyll a; Chl b, chlorophyll b; Chl a/b, chlorophyll a/b ratio; PAR, photosynthetically active radiation; Ti, leaf temperature; RH, relative humidity; VPD, vapor pressure deficit.

The RDA further characterized the multivariate relationships among photosynthetic performance, microenvironmental conditions, pigment traits, and leaf nutrient stoichiometry (**Figure 9**). RDA1 and RDA2 explained 74.62% and 23.82% of the total variation, respectively (cumulatively 98.44%). Leaf N (p < 0.001), leaf P (p < 0.01), N:P (p < 0.05), LT (p < 0.01), VPD (p < 0.01), and Chl a/b (p < 0.05) were significant explanatory variables. Pn was positioned in the positive direction of both RDA1 and RDA2 and was generally aligned with LT, VPD, PAR, Chl a, and Chl a/b. Tr was primarily associated with the positive direction of RDA1, whereas WUE was positioned in the negative RDA1 and positive RDA2 directions. Leaf N and P showed similar ordination directions and were more closely aligned with WUE, whereas N:P was oriented in the positive RDA1 direction.

**Figure 9:**
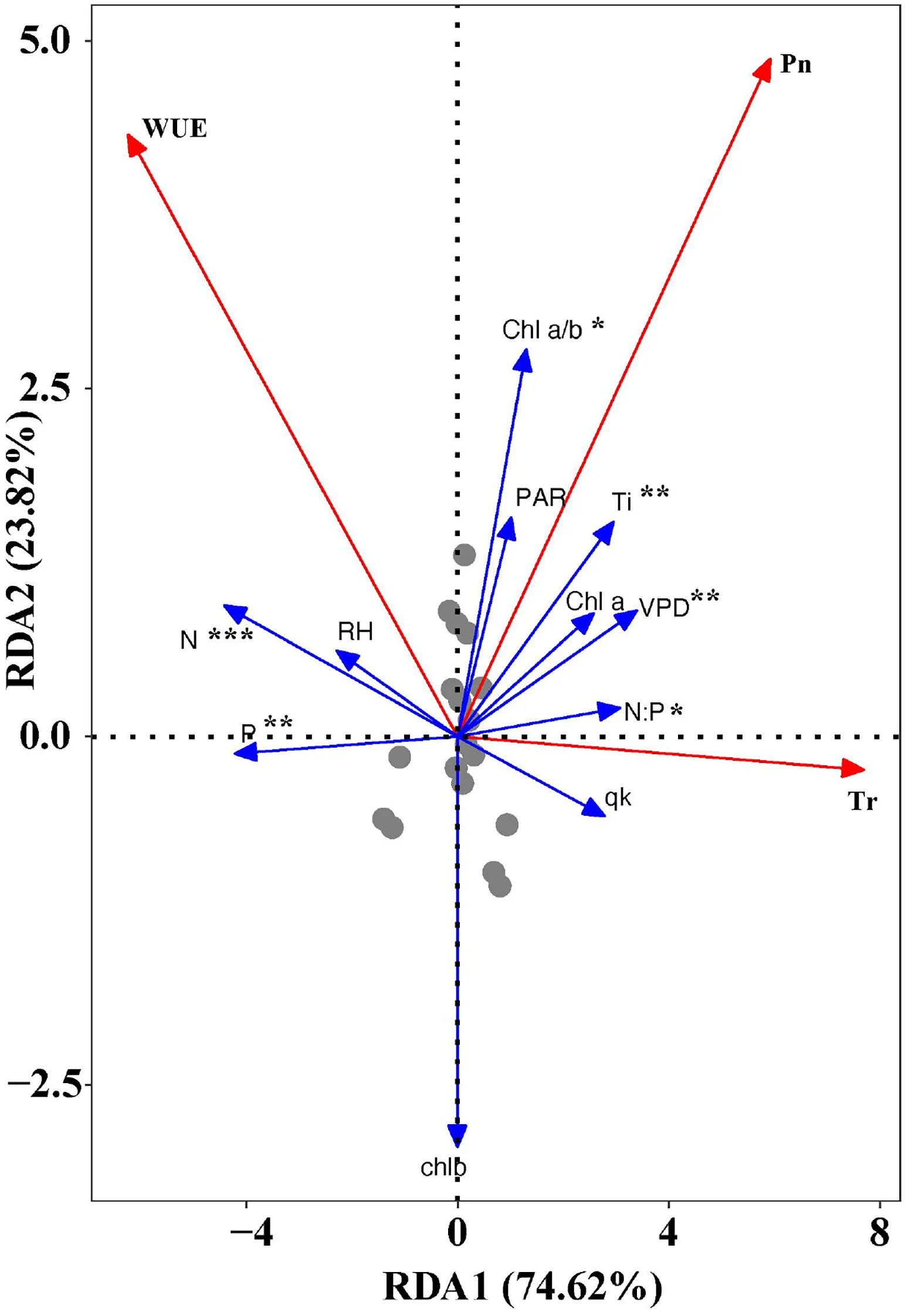
Redundancy analysis (RDA) of the relationships among environmental factors, photosynthetic traits, and leaf N:P stoichiometry of *B. mariqueter*. RDA1 and RDA2 explained 74.62% and 23.82% of the total variation, respectively. Red arrows represent photosynthetic performance variables, blue arrows represent explanatory variables, and gray points represent sampling observations. The direction and length of the arrows indicate the direction and relative strength of associations with the ordination axes, respectively. Asterisks indicate significant effects (p < 0.05, *p < 0.01, and **p < 0.001).

Overall, the random forest, variation partitioning, and RDA results consistently showed that photosynthetic performance was associated with both leaf nutrient stoichiometry and microenvironmental conditions. Leaf N, P, and N:P ratios emerged as important predictors of photosynthetic traits, while the substantial shared explanatory variation among physiological, stoichiometric, and environmental variables indicated that these relationships occurred within an integrated multivariate framework rather than being attributable to a single group of factors.

## 4 Discussion

### 4.1 Photosynthetic strategies of *B. mariqueter* under fluctuating coastal environmental conditions

The photosynthetic characteristics of *B. mariqueter* suggested a physiological strategy suited to the high-irradiance, strongly fluctuating conditions of open coastal tidal flats. Its ability to sustain carbon assimilation under high irradiance is consistent with a habitat characterized by pronounced variation in irradiance, temperature, inundation, and salinity (He et al., 2011; Li et al., 2019b; Wang et al., 2026). Maintaining stomatal opening and transpiration may facilitate CO_2_ diffusion while contributing to transpirational cooling, helping sustain photosynthesis during warm, high-light periods, though at the cost of greater water loss as atmospheric evaporative demand increases. Consistent with this, temperature and water conditions can substantially affect the photosynthetic performance of coastal wetland plants, including *B. mariqueter* (Li et al., 2019a; Liu et al., 2020), whereas thermal acclimation may partly contribute to the maintenance of carbon assimilation under seasonally changing temperature conditions (Liu et al., 2024). Root functional traits can also influence leaf-level physiological responses to changing water availability, suggesting that belowground processes may contribute to the regulation of carbon–water balance under fluctuating environmental conditions (Zhou et al., 2021).

Increased VPD can alter stomatal behavior and leaf gas exchange, thereby affecting the balance between CO_2_ assimilation and transpirational water loss (Hsu et al., 2021; Diao et al., 2024a). Consistent with this mechanism, VPD emerged as an important predictor of both Pn and Tr in random forest analysis. Variation partitioning further showed that microenvironmental factors accounted for a substantial independent fraction of the variation in Pn, while considerable explanatory variation was shared among the microenvironmental, nutrient-stoichiometry, and photosynthetic-pigment variables. These findings indicate that variation in photosynthetic performance cannot be attributed to temperature, VPD, or any single environmental factor alone, but instead reflects responses to multiple covarying environmental and physiological factors.

*B. mariqueter* appeared to dynamically adjust carbon acquisition and water expenditure as environmental conditions changed. Under relatively favorable conditions, transpiration may support CO_2_ uptake and leaf cooling (Diao et al., 2024b; Middleby et al., 2024), whereas increasing atmospheric evaporative demand can increase the water cost of maintaining photosynthesis (Novick et al., 2024). This dynamic balance between carbon gain and water expenditure may represent an important component of the physiological adaptation of *B. mariqueter* to the highly variable conditions of coastal tidal flats (Liu et al., 2024; Zhou et al., 2021).

These physiological responses also have implications for the restoration of *B. mariqueter*. Previous studies have demonstrated the pronounced growth and physiological responses of this species to temperature, waterlogging, salinity, and their interactions (Li et al., 2018; Li et al., 2019b; Liu et al., 2020; Wang et al., 2026). This suggests that maintaining suitable substrate water conditions during periods of high temperature and atmospheric evaporative demand is important for sustaining photosynthetic carbon gain during establishment and restoration. Because *B. mariqueter* has adapted to exposed, high-light, and tidal-flat environments, management practices should avoid unnecessarily reducing light availability. Nevertheless, whether interventions, such as temporary shading or water-level regulation, can improve establishment under extreme summer conditions requires direct experimental evaluation.

### 4.2 Seasonal variation in leaf N and P stoichiometry and implications for nutrient limitation

Seasonal variations in leaf N and P reflect changes in plant growth, nutrient demand, nutrient availability, and environmental conditions, and considerable temporal variations in plant elemental stoichiometry can occur during plant development (Ouyang et al., 2024; Tian et al., 2024). These temporal patterns, therefore, indicate that the relative balance between leaf N and P is seasonally dynamic, and that caution should be exercised in characterizing the nutrient status of *B. mariqueter* using a single growing-season N:P value.

The pronounced seasonal variation in P and N:P ratios suggests that P availability may be an important component of nutrient regulation in *B. mariqueter*. Phosphorus plays a fundamental role in plant energy metabolism and growth, and plant P acquisition and utilization respond strongly to environmental conditions and nutrient availability (Chu et al., 2021; He et al., 2023). Previous experimental evidence has shown that variations in N and P supply can affect the germination and seedling growth of *B. mariqueter* (Wei, 2019), providing independent support for the ecological relevance of nutrient availability in this species. In the present study, leaf P and N:P ratios also emerged as important predictors of photosynthetic performance in the random forest analysis. However, these statistical associations do not indicate that plant growth is limited by P. Although the leaf N:P ratio has been widely used as an empirical indicator of relative N versus P limitation (Wassen et al., 1995; Koerselman and Meuleman, 1996), its interpretation is context dependent and should ideally be supported by additional physiological or experimental evidence. Therefore, our results are more appropriately interpreted as indicating a potentially important role of P availability in seasonal nutrient regulation, rather than demonstrating persistent P limitation.

Nutrient resorption is an important mechanism by which plants conserve and reuse nutrients during leaf senescence, and differences in N and P resorption can provide additional information on plant nutrient-use strategies (Li and Kuai, 2014; Du et al., 2021). At broader scales, nutrient limitations vary considerably among ecosystems and cannot be reliably inferred from a single indicator (Du et al., 2020). Given that present study did not include paired mature and senesced leaves, the N and P resorption efficiencies could not be quantified. Therefore, future studies combining mass-loss-corrected nutrient resorption measurements with factorial N and P addition experiments are needed to determine whether *B. mariqueter* experiences N or P limitation under natural tidal flat conditions.

### 4.3 Coupling between photosynthetic performance and leaf N:P stoichiometry

The relationship between photosynthetic performance and leaf nutrient stoichiometry in *B. mariqueter* is more complex than the simple synchronous response of carbon assimilation to leaf N or P concentrations. Photosynthetic activity was relatively high during the middle of the growing season, whereas leaf N and P concentrations followed distinct seasonal trends. This temporal mismatch suggests that instantaneous or daily carbon assimilation is not determined solely by the absolute concentration of each nutrient. Instead, photosynthetic performance appears to be associated with the balance of leaf nutrient status, together with concurrent changes in environmental conditions and physiological regulation. Nitrogen is closely associated with the photosynthetic apparatus and plant metabolic processes, whereas P plays an essential role in energy metabolism, phosphorylation, and plant growth (Chu et al., 2021; He et al., 2023). More broadly, leaf N and P concentrations are jointly shaped by environmental and biological controls, rather than responding independently to a single environmental driver (Tian et al., 2024). Thus, seasonal changes in leaf N and P may influence photosynthetic capacity; however, their relationships with photosynthetic performance are likely contingent on the environmental conditions under which carbon assimilation occurs.

Multivariate analysis supported this integrated interpretation. The random forest analysis identified leaf nutrient traits as important predictors of photosynthetic performance, although their relative importance differed among the response variables. Leaf P and N:P were particularly important in explaining the variation in Pn, whereas N, P, and N:P contributed strongly to the variation in Tr. N and P were the principal predictors of WUE. These contrasting patterns suggest that leaf nutrient status is associated not only with carbon assimilation, but also with the regulation of water loss and the carbon–water balance. Such multidimensional relationships are consistent with the general principle that plant N and P statuses are closely linked to metabolic activity and growth, while simultaneously responding to environmental conditions (Chu et al., 2021; Ouyang et al., 2024; Tian et al., 2024). Importantly, however, variable importance in the random forest models represents a predictive association rather than direct causal control. Therefore, the strong contribution of nutrient traits should not be interpreted as evidence that variation in photosynthesis is directly caused by N or P limitation.

Variation partitioning further showed that the relationships among photosynthetic performance, nutrient stoichiometry, and environmental conditions strongly overlapped. Photosynthetic pigment traits, leaf nutrient stoichiometry, and microenvironmental factors collectively explained a large proportion of the variation in Pn, Tr, and WUE, and a considerable fraction of the explained variation was shared among the three groups. In particular, microenvironmental factors accounted for a substantial independent fraction of the variation in Pn, indicating that the association between nutrient status and carbon assimilation occurs within a strong environmental context. This interpretation is consistent with evidence that leaf nutrient concentrations are strongly influenced by environmental conditions (Tian et al., 2024), and that photosynthetic performance in *B. mariqueter* responds to temperature, salinity, and hydrological conditions (Li et al., 2018; Li et al., 2019a; Li et al., 2019b; Liu et al., 2020). This shared explanatory structure suggests that seasonal changes in photosynthetic performance and leaf stoichiometry may partly reflect coordinated responses to common environmental and developmental drivers, rather than a simple unidirectional effect of nutrient concentration on photosynthesis.

The RDA provided further evidence for this multidimensional coordination. Leaf N, P, and N:P ratios, together with LT, VPD, and Chl a/b, contributed substantially to the multivariate variation in photosynthetic performance. However, Pn, Tr, and WUE were associated with different combinations of explanatory variables. Pn was more closely aligned with the gradients of LT, VPD, PAR, and pigment characteristics, whereas WUE was more closely associated toward leaf N and P. N:P showed a different ordination direction from N and P, further indicating that nutrient balance and absolute nutrient concentrations may be associated with different components of photosynthetic performance. Such differentiation is ecologically plausible because photosynthetic carbon gain, transpiration, and WUE represent different aspects of plant carbon–water regulation and can respond differently to atmospheric evaporative demand and stomatal regulation (Hsu et al., 2021; Diao et al., 2024a, b). These patterns reinforce the view that photosynthesis–stoichiometry coupling in *B. mariqueter* is multidimensional, rather than reflecting a single linear relationship between leaf nutrient concentration and carbon assimilation.

Collectively, the coupling between photosynthetic performance and leaf N:P stoichiometry in *B. mariqueter* can be interpreted as a coordinated seasonal adjustment of carbon acquisition, water use, pigment characteristics, and nutrient status under changing tidal-flat environmental conditions. Under favorable light and thermal conditions, the species can maintain relatively high carbon assimilation despite substantial seasonal variations in leaf nutrient concentrations, whereas changes in atmospheric evaporative demand and nutrient status are associated with adjustments in transpiration and WUE. Previous studies have demonstrated substantial physiological plasticity of *B. mariqueter* in response to salinity, flooding, temperature, and nutrient conditions (Zhang et al., 2017; Li et al., 2018, 2019; Liu et al., 2020), supporting the view that its performance in tidal flat habitats depends on the integration of multiple resources and environmental dimensions. This coordination may allow *B. mariqueter* to balance its carbon gain, water expenditure, and nutrient use throughout the growing season. Nevertheless, because the present analyses were based on observational relationships, the direction of causality among environmental conditions, nutrient stoichiometry, and photosynthetic performance could not be established. Manipulative experiments that combine N and P availabilities with water and temperature treatments are required to determine the causal mechanisms underlying this coupling.

Thus, photosynthesis–stoichiometry coupling in *B. mariqueter* appears to represent an integrated and environmentally embedded coordination of carbon acquisition, water use, and nutrient status, rather than a simple direct control of photosynthesis by leaf N or P concentrations.

## 5 Conclusion

*B. mariqueter* exhibited a photosynthetic strategy suited to the high-light and seasonally variable conditions of coastal tidal flats. Photosynthetic performance varied substantially over the growing season and was closely associated with changes in light availability, atmospheric evaporative demand, water-use processes, and leaf nutrient status. The broadly coordinated diurnal dynamics of Pn and Tr, together with the partial seasonal divergence between daily carbon assimilation and transpiration, indicate that carbon gain and water loss are dynamically regulated rather than strictly synchronized throughout the growing season.

Leaf N and P concentrations and the N:P ratio also showed pronounced and asynchronous seasonal variation. Random forest analysis identified leaf N, P, and N:P, together with VPD and other microenvironmental variables, as important predictors of Pn, Tr, and WUE. Variation partitioning further showed substantial shared explanatory variation among photosynthetic–pigment traits, leaf nutrient stoichiometry, and microenvironmental factors, while RDA demonstrated that different components of photosynthetic performance were associated with different combinations of environmental and nutrient variables. These results indicate that photosynthetic performance and leaf nutrient stoichiometry are linked within an integrated environmental–physiological framework rather than through a simple direct relationship between leaf N or P concentration and carbon assimilation. Although P availability may play an important role in seasonal nutrient regulation, the present study does not provide sufficient evidence to diagnose persistent P limitation.

These findings have practical implications for the restoration and cultivation of *B. mariqueter*. Restoration sites should provide adequate light while maintaining sufficient substrate water availability during periods of high temperature and atmospheric evaporative demand. Under extreme summer conditions, temporary moderate shading may be considered to reduce excessive evaporative stress, although this management approach requires experimental verification. Leaf nutrient status, particularly P availability and N:P balance, should also be considered when evaluating restoration substrates, but nutrient supplementation should be based on site-specific nutrient conditions rather than applied indiscriminately. Future experiments combining water, temperature, and N- and P-addition treatments will be necessary to identify the environmental and nutritional conditions that optimize establishment and population expansion of *B. mariqueter*.

## Data Availability

The raw data supporting the conclusions of this article will be made available by the authors, without undue reservation.
